# Molecular Diagnosis of Orthopedic-Device-Related Infection Directly from Sonication Fluid by Metagenomic Sequencing

**DOI:** 10.1128/JCM.00462-17

**Published:** 2017-07-25

**Authors:** Teresa L. Street, Nicholas D. Sanderson, Bridget L. Atkins, Andrew J. Brent, Kevin Cole, Dona Foster, Martin A. McNally, Sarah Oakley, Leon Peto, Adrian Taylor, Tim E. A. Peto, Derrick W. Crook, David W. Eyre

**Affiliations:** a Nuffield Department of Clinical Medicine, University of Oxford, John Radcliffe Hospital, Oxford, United Kingdom; b Bone Infection Unit, Nuffield Orthopaedic Centre, Oxford University Hospitals NHS Foundation Trust, Oxford, United Kingdom; c Microbiology Laboratory, John Radcliffe Hospital, Oxford University Hospitals NHS Foundation Trust, Oxford, United Kingdom; d Department of Infectious Diseases and Microbiology, Royal Sussex County Hospital, Brighton, United Kingdom; e Public Health England, Microbiology, Royal Sussex County Hospital, Brighton, United Kingdom; f National Institute for Health Research Oxford Biomedical Research Centre, John Radcliffe Hospital, Oxford, United Kingdom; Medical College of Wisconsin

**Keywords:** diagnosis, metagenomic sequencing, orthopedic device infection, prosthetic joint infection

## Abstract

Culture of multiple periprosthetic tissue samples is the current gold standard for microbiological diagnosis of prosthetic joint infections (PJI). Additional diagnostic information may be obtained through culture of sonication fluid from explants. However, current techniques can have relatively low sensitivity, with prior antimicrobial therapy and infection by fastidious organisms influencing results. We assessed if metagenomic sequencing of total DNA extracts obtained direct from sonication fluid can provide an alternative rapid and sensitive tool for diagnosis of PJI. We compared metagenomic sequencing with standard aerobic and anaerobic culture in 97 sonication fluid samples from prosthetic joint and other orthopedic device infections. Reads from Illumina MiSeq sequencing were taxonomically classified using Kraken. Using 50 derivation samples, we determined optimal thresholds for the number and proportion of bacterial reads required to identify an infection and confirmed our findings in 47 independent validation samples. Compared to results from sonication fluid culture, the species-level sensitivity of metagenomic sequencing was 61/69 (88%; 95% confidence interval [CI], 77 to 94%; for derivation samples 35/38 [92%; 95% CI, 79 to 98%]; for validation samples, 26/31 [84%; 95% CI, 66 to 95%]), and genus-level sensitivity was 64/69 (93%; 95% CI, 84 to 98%). Species-level specificity, adjusting for plausible fastidious causes of infection, species found in concurrently obtained tissue samples, and prior antibiotics, was 85/97 (88%; 95% CI, 79 to 93%; for derivation samples, 43/50 [86%; 95% CI, 73 to 94%]; for validation samples, 42/47 [89%; 95% CI, 77 to 96%]). High levels of human DNA contamination were seen despite the use of laboratory methods to remove it. Rigorous laboratory good practice was required to minimize bacterial DNA contamination. We demonstrate that metagenomic sequencing can provide accurate diagnostic information in PJI. Our findings, combined with the increasing availability of portable, random-access sequencing technology, offer the potential to translate metagenomic sequencing into a rapid diagnostic tool in PJI.

## INTRODUCTION

Prosthetic joint infections (PJI) are a devastating and difficult-to-treat complication of joint replacement surgery. Although the relative incidence of PJI is low (0.8% of knee and 1.2% of hip replacements across Europe) (1), given the increasing numbers of arthroplasties performed worldwide, PJI are a significant health care burden and cause of expense. For individual patients, PJI often require multiple surgeries, intensive, long-term antimicrobial therapy, and a prolonged period of rehabilitation. Fast, accurate, and reliable diagnosis of PJI is necessary to inform treatment choices, particularly for antibiotic-resistant organisms. Culture of multiple periprosthetic tissue (PPT) samples remains the gold standard for microbial detection (2–4). However, culture can be relatively insensitive, with only 65% of causative bacteria detected in infections even when multiple PPT samples are collected (2, 5). Infections with fastidious organisms or infections in a patient who has received prior antimicrobial treatment are often culture negative.

Culture of sonication fluid from explanted prostheses may improve microbiological yield in PJI by disrupting the bacterial biofilm. Since sonication was first applied to explanted hip prostheses in 1998 (6), several clinical studies have reported the improved sensitivity of sonication fluid culture over PPT culture for the diagnosis of hip, knee, and shoulder PJI (7, 8), and sonication has been adopted by many centers, either alone or in combination with PPT culture. Additionally, several molecular assays have been investigated to improve the sensitivity of PJI diagnosis. PCR assays using DNA extracted from sonication fluid (9, 11, 12, 40) have reported sensitivities ranging from 70% to 96%. However, this approach can identify only pathogens in a predefined multiplex panel and thus may miss atypical or rare pathogens not targeted in the assay design. Other studies identify pathogens by amplification and sequencing of the universal bacterial 16S rRNA gene (10, 13, 14). A drawback of these methods is the potential for generating false-positive results from contaminating bacterial DNA.

The potential of high-throughput sequencing as a diagnostic tool for infectious diseases is widely recognized (15–17). Metagenomic sequencing offers the possibility to detect all DNA in a clinical sample, which can then be compared to reference genome databases to identify pathogens. Additionally, a profile of common laboratory and kit contaminants can be generated from negative controls sequenced concurrently, and this information can be taken into account (18, 19). In addition to diagnostic data, whole-genome sequencing can also simultaneously provide characterization of infection outbreaks (20, 21), track transmission (22–24), and predict antimicrobial resistance (25–28). At present, most whole-genome sequencing studies rely on sequencing DNA extracted from a cultured isolate, and extending these approaches to metagenomic sequencing data is an active area of research. An advantage offered by sequencing is the speed at which it can deliver genetic information (29) compared to that of traditional microbiological culture and antimicrobial susceptibility testing, which can take days to weeks depending on the pathogen. By removing a culture step and sequencing directly from clinical samples, the time taken to diagnosis can be reduced further (30), and pathogens not identified by conventional methods can be detected (31–33). Here, we investigated if metagenomic sequencing of total DNA extracts obtained directly from sonication fluid can provide an alternative rapid and sensitive tool for diagnosis of PJI, without the need for a culture step.

## RESULTS

**FIG 1:**
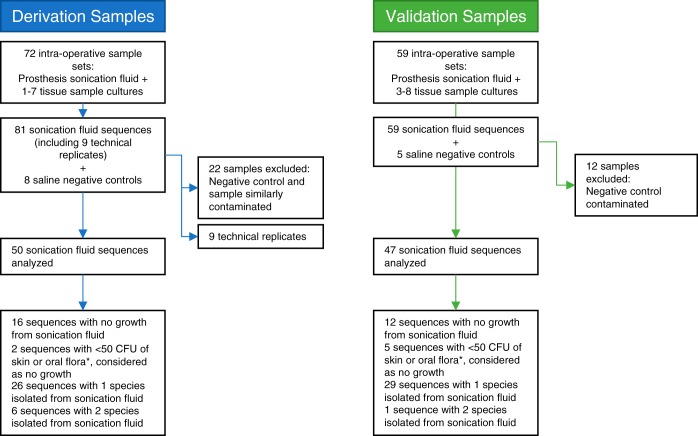
Study samples and quality control. Sequences with <50 CFU (*) represent Staphylococcus epidermidis, other coagulase-negative staphylococci, viridans group streptococci, and Propionibacterium acnes.

A total of 131 sonication fluid samples from patients undergoing revision arthroplasty or removal of other orthopedic devices were aerobically and anaerobically cultured and subjected to metagenomic sequencing (Fig. 1). Additionally, a median of 5 (interquartile range [IQR], 4 to 5; range, 1 to 8) PPT samples were cultured from each patient. From the first 72 sonication fluid samples sequenced, 22 samples from six batches were excluded as these samples and negative controls from the same batches showed similar contamination levels (see Materials and Methods) (Fig. 1). The remaining 50 samples, the derivation set, were used to determine optimal sequence thresholds for identifying true infection. Of 59 subsequently sequenced validation samples, 12 from a single batch were excluded as the negative control was contaminated with Propionibacterium acnes, leaving 47 validation samples sequenced in batches with uncontaminated negative controls. In the 97 samples analyzed, Staphylococcus aureus, isolated from 22% of sonication fluids and 29% PPTs, and Staphylococcus epidermidis, isolated from 13% of sonication fluids and 25% of PPTs, were the two most frequently cultured species (Table 1).

**TABLE 1:** Summary of species observed in culture of sonication fluid and periprosthetic tissue from 97 cases, presented by joint/implant type

Species	No. of patients positive by sonication fluid (no. positive by PPT)^*a*^					
	Ankle (*n* = 6)	Hip (*n* = 32)	Knee (*n* = 42)	Metalwork (*n* = 14)^*b*^	Shoulder (*n* = 3)	Total (*n* = 97)
Staphylococci						
Staphylococcus aureus	0 (1)	5 (9)	10 (11)	5 (6)	1 (1)	21 (28)
Staphylococcus condimenti			1 (0)			1 (0)
Staphylococcus epidermidis	0 (1)	6 (10)	7 (12)	0 (1)	0 (1)	13 (25)
Staphylococcus lugdunensis	0 (1)	1 (1)		1 (1)		2 (3)
Coagulase-negative Staphylococcus		0 (3)	1 (2)	0 (1)	1 (0)	2 (6)
Streptococci						
Streptococcus agalactiae			2 (2)			2 (2)
Streptococcus dysgalactiae		0 (1)				0 (1)
Streptococcus oralis		0 (2)				0 (2)
Streptococcus pneumoniae		1 (1)				1 (1)
Enterococci						
Enterococcus faecalis		3 (4)	2 (2)	0 (1)		5 (7)
Enterococcus faecium		0 (1)	3 (3)			3 (4)
Enterobacteriaceae						
Citrobacter koseri		1 (1)				1 (1)
Citrobacter species			1 (1)			1 (1)
Enterobacter cloacae	1 (1)		1 (1)			2 (2)
Escherichia coli		1 (2)				1 (2)
Klebsiella oxytoca				0 (1)		0 (1)
Klebsiella pneumoniae			1 (1)			1 (1)
Morganella morganii			2 (2)			2 (2)
Proteus mirabilis		0 (1)	1 (1)			1 (2)
Serratia marcescens			1 (1)			1 (1)
Corynebacteria						
Corynebacterium amycolatum		0 (1)				0 (1)
Corynebacterium aurimucosum		0 (1)				0 (1)
Corynebacterium propinquum				0 (1)		0 (1)
Corynebacterium striatum		0 (3)		0 (1)		0 (4)
Other						
Aeromonas species				0 (1)		0 (1)
Aeromonas hydrophila				1 (0)		1 (0)
Arcanobacterium haemolyticum		1 (0)				1 (0)
Bacillus species	0 (1)	1 (2)				1 (3)
Finegoldia magna				1 (0)		1 (0)
Gemella morbillorum		1 (1)	1 (1)			2 (2)
Granulicatella adiacens				0 (1)		0 (1)
Micrococcus luteus				0 (1)		0 (1)
Mycobacterium fortuitum			0 (1)			0 (1)
Propionibacterium acnes		1 (1)				1 (1)
Propionibacterium spp.		0 (1)				0 (1)
Pseudomonas aeruginosa	1 (2)	0 (2)		1 (2)		2 (6)
No growth	5 (3)	12 (7)	11 (6)	6 (4)	1 (1)	35 (21)
Total no. of species isolated	7 (10)	34 (55)	45 (47)	15 (22)	3 (3)	104 (137)^*c*^

a Results are reported for patients with ≥1 isolate of the indicated species from sonication fluid and PPT from the indicated sample source. Sonication fluid cultures were considered positive if >50 CFU/ml was isolated or if <50 CFU/ml of a virulent organism (i.e., not skin or oral flora) was isolated. *n*, number of patients.

b Metalwork comprises plates and/or screws from tibia (*n* = 3), femur (*n* = 4), spine (*n* = 2), foot (*n* = 2), humerus (*n* = 1), ankle (*n* = 1), and ulna (*n* = 1).

c The numbers in the table reflect the fact that some samples were positive for more than one organism.

The 97 sonication fluid samples passing sequencing quality-control checks were obtained predominantly from knee (42/97, or 43%) and hip (32, or 33%) PJI, with other samples from ankle (6, or 6%) and shoulder (3, or 3%) PJI and other orthopedic device infections (14, or 14%) (Table 1). The median sonication fluid volume was 200 ml (IQR, 100 to 400 ml; range, 15 to 400 ml) (see Table S1 in the supplemental material). On culture, 35 (36%) sonication fluid samples had no growth or less than 50 CFU of an organism not considered to be highly pathogenic (skin and oral flora), 55 (57%) samples had a single organism isolated, and 7 (7%) samples had two organisms isolated. Greater than 10^6^ reads were achieved in 91/97 (94%) samples. Taxonomic classification by Kraken identified a median of 0.07% (IQR, 0.01 to 0.41%; range, <0.01% to 24.0%) of reads as bacterial, with <1% of bacterial reads in 84/97 (87%) samples. Human reads accounted for >90% of reads in 94/97 (97%) of samples. Six test samples were processed with and without the NEBNext microbiome DNA enrichment kit. Use of the kit did not reduce the amount of human DNA sequenced. The mean proportion of reads classified as human was 98.4% with the enrichment kit and 98.2% without it (*P* = 0.06) (Table S2).

**FIG 2:**
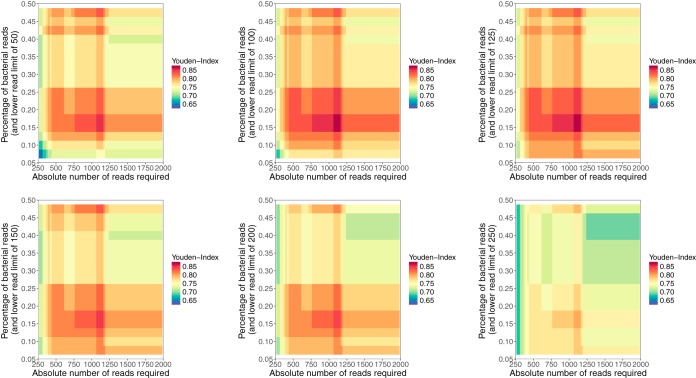
Sequencing data filtering calibration heat maps. Two thresholds (threshold 1 and threshold 2) and three parameters (parameter a, parameter b, and parameter c) were used to determine true infection. Samples meeting either threshold were determined to be true infection. The final parameter values were chosen by maximizing the Youden index, calculated as follows: (sensitivity + specificity) − 1. For threshold 1, samples with more reads from a given species than an upper-read cutoff (parameter a; plotted on each *x* axis) were included. For threshold 2, samples with more species-specific reads than a lower-read cutoff (parameter b; the six panels show six different values for parameter b: 50, 100, 125, 150, 200, and 250, which are indicated within each *y*-axis title) and with the percentage of species-specific reads as a proportion of all bacterial reads present above a percentage cutoff (parameter c, plotted on each *y* axis) were included.

Optimal thresholds for determining if samples contained low-level contamination or true infection were determined by numerical optimization, choosing thresholds that maximized the sensitivity and specificity of sequencing (Fig. 2). The final thresholds chosen to determine the presence of true infection were ≥1,150 reads from a single species or ≥125 reads from a single species if ≥15% of the total bacterial reads also belonged to that same species.

Samples extracted and sequenced as replicates showed good reproducibility. In four duplicate and one triplicate culture-positive sample, the same species was recovered by sequencing on all occasions (samples 164, 171, 182, 183, and 193). A single replicate, 182a, had an additional, likely contaminating, species identified (not found in sonication fluid or PPT culture). A single culture-negative sample (sample 176) was processed in triplicate. One of the three replicates (176a) had an apparent contaminating species identified (also not found in sonication fluid or PPT culture).

Table 2 compares sonication culture results with metagenomic sequencing findings, applying our sequencing data thresholds. PPT culture results and the consensus microbiology diagnosis based on both sonication and PPT samples are also given for comparison. Compared to sonication fluid culture, metagenomic sequencing had an overall species-level sensitivity of 61/69 (88%; 95% CI, 77 to 94%). Sensitivity was 35/38 (92%; 95% CI, 79 to 98%) in the derivation samples and 26/31 (84%; 95% CI, 66 to 95%) in the validation samples. Three samples were identified to the genus level only. Hence, overall genus-level sensitivity was 64/69 (93%; 95% CI, 84 to 98%). Of the other five samples where the species cultured was not identified on sequencing, two samples cultured a coagulase-negative Staphylococcus not identified on tissue culture, one sample was polymicrobial (where several species found in sonication fluid or tissue were identified, but not all), and the remaining two samples were negative for a pathogen found in sonication fluid and tissue.

**TABLE 2:** Comparison of species identified from sonication fluid and PPT culture with species identified from metagenomic sequencing reads for all samples passing thresholds for analysis in the derivation and validation data sets^*a*^

Sample group and no.	Sonication species	Sonication CFU count	Tissue culture species	No. of positive tissue samples/total no. of samples	Sequencing species	No. of reads	% bacterial reads	False result^*b*^
Derivation set (*n* = 50)								
164	S. epidermidis	>490	S. epidermidis	3/5	S. epidermidis	2,716	81	
171	S. epidermidis	>490	S. epidermidis	2/5	S. epidermidis	3,154	79	
182	E. faecium	100–240	E. faecium	6/6	E. faecium	144	43	
183	S. epidermidis	>490	S. epidermidis	4/5	S. epidermidis	3,362	87	
193	S. aureus	>490	S. aureus	5/5	S. aureus	360,718	97	
	S. condimenti	>490						FN; not in database; genus only
198	S. epidermidis	>490	S. epidermidis	3/5	S. epidermidis	228	52	
208	E. faecalis	>490	E. faecalis	5/5	E. faecalis	14,486	31	
	E. coli	250–490	E. coli	4/5	E. coli	6,503	14	
213	S. aureus	>490	S. aureus	5/5	S. aureus	167	80	
219	S. lugdunensis	>490	S. lugdunensis	3/4	S. lugdunensis	411	27	
			C. propinquum	4/4	A. xylosoxidans	722	47	FP
			S. epidermidis	1/4				
223	S. aureus	>490	S. aureus	4/5	S. aureus	7,504	95	
229	S. aureus	>490	S. aureus	1/2	S. aureus	6,038	98	
249	P. acnes	>490	P. acnes	4/5	P. acnes	108,940	100	
259	S. epidermidis	>490	S. epidermidis	3/4	S. epidermidis	749	86	
289	S. aureus	250–490	S. aureus	5/5	S. aureus	2,105	94	
296	S. marcescens	250–490	S. marcescens	4/4	S. marcescens	590	60	
312	C. koseri	>490	C. koseri	4/5	C. koseri	221,516	95	
329	M. morganii	>490	M. morganii	6/6	M. morganii	18,553	95	
335	M. morganii	100–240	M. morganii	3/5	M. morganii	3,555	94	
352	Bacillus spp.	100–240	Bacillus spp.	2/5	Bacillus spp.	1,109		FN; genus only
354	A. haemolyticum	>490	S. aureus	2/6	A. haemolyticum	11,182	72	
	E. faecalis	>490	E. faecalis	4/6	E. faecalis	1,173	8	
			S. oralis	1/6	F. nucleatum	1,156	7	FP; plausible anaerobe
			CoNS	5/6				
			P. aeruginosa	1/6				
			C. striatum	1/6				
			S. epidermidis	2/6				
361	F. magna	>490	No growth	0/6	F. magna	3,674	95	
366	K. pneumoniae	>490	K. pneumoniae	4/5	K. pneumoniae	8,981	25	
369	E. cloacae	>490	E. cloacae	4/5	E. cloacae	2,502	11	
	P. aeruginosa	100–240	P. aeruginosa	5/5	P. aeruginosa	1,192	5	
			S. epidermidis	4/5	V. parvula	14,801	65	FP; plausible anaerobe
			S. lugdunensis	1/5				
371	S. epidermidis	>490	S. epidermidis	3/3	S. epidermidis	4,998	87	
			CoNS	1/3				
373	E. faecalis	>490	E. faecalis	1/5	E. faecalis	1,234	38	
	S. epidermidis	100–240	S. epidermidis	3/5	S. epidermidis	616	19	
376	E. cloacae	>490	E. cloacae	4/4	E. cloacae	122,622	95	
	CoNS	>490						FN; probable plate contaminant
382	S. aureus	<50	S. aureus	4/4	S. aureus	440	50	
			S. dysgalactiae	2/4				
384	S. epidermidis	>490	S. epidermidis	2/4	S. epidermidis	1,751	85	
399	S. aureus	Not recorded	S. aureus	2/5	S. aureus	1,955	97	
404	S. aureus	>490	S. aureus	4/6	S. aureus	2,257	39	
			C. striatum	5/6				
			E. coli	2/6				
408	S. aureus	>490	S. aureus	4/4	S. aureus	368	87	
410	S. aureus	100–240	S. aureus	4/4	S. aureus	235	27	
			CoNS	1/4	C. jeikeium	401	46	FP
362	P. acnes	<50	No growth	0/1				
370	P. acnes	<50	No growth	0/4				
176	No growth		S. aureus	1/4				
			P. aeruginosa	3/4				
346	No growth		S. aureus	3/5				
			M. fortuitum	1/5				
359	No growth		S. epidermidis	1/4	P. acnes	464	24	FP; P. acnes
372	No growth		S. aureus	4/4	P. acnes	3,874	51	FP; P. acnes
			G. adiacens	1/4				
375	No growth		S. epidermidis	1/5	P. acnes	5,686	75	FP; P. acnes
379	No growth		S. aureus	3/5				
			CoNS	1/5				
389	No growth		S. epidermidis	2/5				
			Bacillus spp.	1/5				
341	No growth		No growth	0/3	S. aureus	153	42	FP; prior flucloxacillin exposure; plausible pathogen
358	No growth		No growth	0/3				
364	No growth		No growth	0/4				
365	No growth		No growth	0/1	P. acnes	318	23	FP; P. acnes
368	No growth		No growth	0/4	R. pickettii	3,146	40	FP
					E. cloacae	2,629	33	FP
374	No growth		No growth	0/4				
383	No growth		No growth	0/4				
388	No growth		No growth	0/3				
391	No growth		No growth	0/4				
Validation set (*n* = 47)								
256	G. morbillorum	>490	G. morbillorum	6/6	G. morbillorum	784	72	
397	S. epidermidis	>490	S. epidermidis	5/5	S. epidermidis	6,717	94	
400	A. hydrophila	>490	Aeromonas spp.	3/4				FN
	S. aureus	100–240	S. aureus	4/4	S. aureus	6,547	5	
			P. aeruginosa	2/4	P. aeruginosa	86,920	68	FP; in tissue
			K. oxytoca	1/4	K. oxytoca	1,238	1	FP; in tissue
					F. magna	15,606	12	FP; plausible anaerobe
			E. faecalis	1/4	E. faecalis	1,303	1	FP; in tissue
405	S. lugdunensis	>490	S. lugdunensis	6/6	S. lugdunensis	311	96	
406	E. faecium	250–490	E. faecium	2/3				FN
409	S. agalactiae	>490	S. agalactiae	5/5	S. agalactiae	2,556	93	
423	S. aureus	>490	S. aureus	4/4	S. aureus	15,479	98	
426	S. aureus	250–490	S. aureus	2/4	S. aureus	11,981	89	
430	S. pneumoniae	>490	S. pneumoniae	5/5	S. pneumoniae	5,697	82	
442	E. faecium	>490	E. faecium	5/5	E. faecium	1,689	68	
450	S. aureus	>490	S. aureus	5/6	S. aureus	2,584	98	
			Propionibacterium spp.	1/6				
459	S. agalactiae	>490	S. agalactiae	5/5	S. agalactiae	114,212	93	
465	S. aureus	>490	S. aureus	4/4	S. aureus	1,171	97	
468	S. aureus	>490	S. aureus	3/3	S. aureus	676	93	
473	E. faecalis	250–490	E. faecalis	4/4	E. faecalis	228	73	
474	S. epidermidis	250–490	C. striatum	2/5				FN; genus only
			S. aureus	1/5				
			S. epidermidis	3/5				
480	S. epidermidis	250–490	S. epidermidis	5/5	S. epidermidis	557	80	
482	S. epidermidis	>490	S. epidermidis	5/5	S. epidermidis	1,327	88	
483	S. aureus	100–240	No growth	0/5	S. aureus	444	85	
485	G. morbillorum	>490	G. morbillorum	3/4	G. morbillorum	123,300	18	
			S. oralis	1/4	P. micra	508,822	76	FP; plausible anaerobe
			S. aureus	1/4	S. equi	16,580	2	FP
			C. amycolatum	1/4	S. anginosus	8,019	1	FP; plausible anaerobe
			P. mirabilis	1/4				
486	E. faecalis	>490	E. faecalis	5/5	E. faecalis	3,904	43	
			S. epidermidis	4/5				
487	S. aureus	<50	S. aureus	2/4	S. aureus	121,284	98	
489	S. aureus	100–240	S. aureus	2/4	S. aureus	858	95	
			S. epidermidis	1/4				
498	S. aureus	<50	S. aureus	4/5	S. aureus	135	88	
504	S. aureus	>490	S. aureus	7/7	S. aureus	3,229	97	
507	P. mirabilis	<50	P. mirabilis	2/5	P. mirabilis	184	15	
					M. morganii	981	83	FP
511	P. aeruginosa	Not recorded	P. aeruginosa	3/6				FN
					P. acnes	1,377	69	FP; P. acnes
513	Citrobacter spp.	<50	Citrobacter spp.	2/5	C. koseri	1,133	87	
514	S. epidermidis	>490	S. epidermidis	5/5	S. epidermidis	11,803	91	
516	CoNS	100–240	No growth	0/4				FN; probable plate contaminant
414	S. epidermidis	<50	S. epidermidis	5/5	S. epidermidis	1,194	91	FP; low sonication count; in tissue
490	S. epidermidis	<50	No growth	0/5				
497	S. vestibularis	<50	C. striatum	4/4				
503	CoNS	<50	No growth	0/5				
512	S. epidermidis	<50	S. epidermidis	5/5				
475	No growth		M. luteus	1/4	S. dysgalactiae	156	37	FP; prior flucloxacillin exposure; plausible pathogen
476	No growth		P. aeruginosa	4/4				
			S. aureus	3/4				
478	No growth		CoNS	1/4				
496	No growth		Bacillus spp.	1/4				
502	No growth		C. aurimucosum	2/4	C. aurimucosum	2,379	42	FP; in tissue
			S. epidermidis	4/4	S. epidermidis	1,336	24	FP; in tissue
			E. faecium	3/4				
			CoNS	2/4				
510	No growth		S. epidermidis	2/4	S. epidermidis	290	26	
					P. acnes	232	21	FP; P. acnes
515	No growth		E. faecalis	1/7	P. acnes	873	34	FP; P. acnes
472	No growth		No growth	0/4				
505	No growth		No growth	0/5				
506	No growth		No growth	0/6				
508	No growth		No growth	0/8				
509	No growth		No growth	0/5				

a Abbreviations for species not mentioned in the text are as follows: A. hydrophila, Aeromonas hydrophila; C. koseri, Citrobacter koseri; C. aurimucosum, Corynebacterium aurimucosum; C. jeikeium, Corynebacterium jeikeium; C. propinquum, Corynebacterium propinquum; C. striatum, Corynebacterium striatum; E. cloacae, Enterobacter cloacae; E. faecalis, Enterococcus faecalis; E. faecium, Enterococcus faecium; G. morbillorum, Gemella morbillorum; K. oxytoca, Klebsiella oxytoca; M. luteus, Micrococcus luteus; M. morganii, Morganella morganii; M. fortuitum, Mycobacterium fortuitum; P. mirabilis, Proteus mirabilis; P. aeruginosa, Pseudomonas aeruginosa; R. pickettii, Ralstonia pickettii; S. lugdunensis, Staphylococcus lugdunensis; S. agalactiae, Streptococcus agalactiae; S. equi, Streptococcus equi; S. oralis, Streptococcus oralis; S. vestibularis, Streptococcus vestibularis; CoNS, coagulase-negative Staphylococcus species. See also Table S1 for genus details.

b FN, false-negative result; FP, false-positive result.

Overall species-level specificity was 78/97 (80%; 95% CI, 71 to 88%). However, of 19 samples where additional species were identified on sequencing compared to results with sonication culture, three (samples 400, 414, and 502) had the same species found in tissue culture but not in sonication fluid (or the level was <50 CFU). Four samples (samples 354, 369, 400, and 485) had plausible anaerobic causes of infection (Fusobacterium nucleatum, Veillonella parvula, Finegoldia magna, and Parvimonas micra [identified alongside Streptococcus anginosus]). Samples 341 and 475 contained S. aureus and Streptococcus dysgalactiae DNA, respectively, both in patients who had received prior flucloxacillin, and no microbiological diagnosis was reached based on culture. However, 12 samples (including sample 485) had other species found on sequencing not otherwise identified. In some cases these were clearly laboratory contaminants, e.g., sample 219 contained Achromobacter xylosoxidans reads, and an A. xylosoxidans culture-positive sample was sequenced in the same batch from a concurrent study. Notably P. acnes was a common contaminant occurring in 7/97 (7%) samples overall. Adjusting for plausible fastidious causes of infection, species found in concurrently obtained PPT samples, and prior antibiotics, i.e., assuming these samples were actually genuinely positive for the species found on sequencing, species-level specificity was 85/97 (88%; 95% CI, 79 to 93%) overall, 43/50 (86%; 95% CI, 73 to 94%) in the derivation samples, and 42/47 (89%; 95% CI, 77 to 96%) in the validation samples.

**FIG 3:**
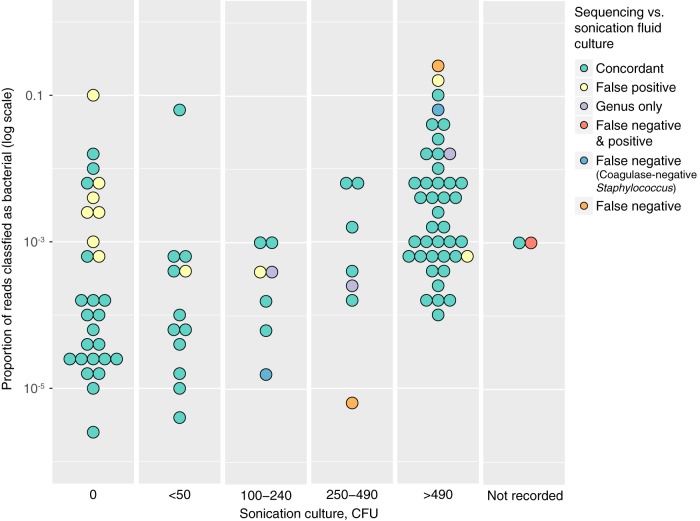
Sonication culture and sequencing comparison. The proportion of sequencing reads classified as bacterial is shown on the *y* axis on a log scale, and the number of CFU from sonication fluid culture is shown on the *x* axis. Markers are colored by the concordance of sonication fluid culture and sequencing. A single marker is shown per patient sample. Where only one of several species isolated was found by sequencing, this is shown as a false-negative. Similarly, any sample with one or more false-positive species identified by sequencing is shown as false positive. False-negative results where a coagulase-negative Staphylococcus was cultured from sonication fluid but not found in tissue samples or on sequencing are shown separately, as are samples identified only to the genus level by sequencing. Results were very similar if absolute numbers of bacterial reads were plotted on the *y* axis instead.

Figure 3 shows the relationship between the proportion of sequence reads obtained that were classified as bacterial, the sonication fluid culture CFU counts, and the concordance between sonication fluid culture and sequencing. Sequencing false-positive results were more likely when cultures were negative.

More simplistic thresholds based on a single cutoff for determining true infection performed less well. Within the derivation samples, using a single cutoff for the proportion of bacterial reads from a given species, irrespective of the absolute numbers of bacterial reads present, the optimal cutoff value was 25%. Using this threshold, species-level sensitivity was 57/69 (83%) and adjusted specificity was 80/97 (82%). Similarly, if only a single absolute read number cutoff is used, the optimal value is 410 reads from a single species, and sensitivity is 54/69 (78%) and adjusted specificity is 87/97 (90%).

Sequencing results were also compared to a consensus microbiology diagnosis based on guidelines of the Infectious Diseases Society of America (IDSA) (4), considering any species isolated twice or any virulent species isolated as a cause of infection, combining sonication and PPT culture results (Table S1). These results showed that 66/97 (68%) samples demonstrated complete agreement between the consensus species list from culture and sequencing, 14/97 (14%) samples had a partial match with at least one species found on culture also found on sequencing, 15/97 (15%) samples had none of the species cultured found on sequencing, and 2/97 (2%) samples had a plausible additional species found on sequencing not found on culture. The sensitivity of sonication fluid sequencing compared to that of combined sonication fluid and PPT culture was 67/99 (68%), and specificity was 80/97 (82%); as above, specificity, adjusting for plausible fastidious causes of infection and prior antibiotics, was 85/97 (88%).

## DISCUSSION

Diagnosis of PJI by culture of sonication fluid and PPT is not always conclusive and may take up to 10 to 14 days for slow-growing organisms. Here, we assess, for the first time, the use of metagenomic sequencing of total DNA extracts obtained directly from sonication fluid in the diagnosis of PJI. We developed a novel filtering strategy to ensure that low-level contaminating DNA is successfully ignored while infections are detected accurately. Compared to sonication fluid culture, metagenomic sequencing achieved a species-level sensitivity of 88% and specificity of 88%, after adjusting for plausible fastidious causes of infection, species found in concurrently obtained PPT samples, and prior antibiotic use. Importantly we demonstrated similar performance of our method and a filtering algorithm in the subset of samples that formed an independent validation set, with sensitivity of 84% and adjusted specificity of 89%.

Sequencing failed to identify an organism cultured from sonication fluid for eight samples. For two samples, a coagulase-negative Staphylococcus was cultured but only from sonication fluid and not from tissue samples. These isolates, therefore, could plausibly have been plate contaminants and not present in the DNA sequenced. For three other samples, identification to the genus level was possible. One sample contained Staphylococcus condimenti, which was not included in our custom Kraken database, highlighting the limitation that, despite including 2,786 bacterial genomes, this approach is only as good as the database that is used. Another sample was identified as a Bacillus spp. both on culture and by sequencing, and the third was identified by sequencing as Staphylococcus spp. in the context of a mixed Staphylococcus infection. For the three remaining samples, sequencing failed to identify a pathogen found on culture.

Sequencing was also able to detect potential pathogens not identified by culture of sonication fluid. For three samples we identified additional species from sequencing that were supported by the tissue culture findings, suggesting that in some settings sequencing may be more sensitive than sonication fluid culture alone without PPT culture although this might also be explained by the additional centrifugation prior to sequencing to ensure sufficient DNA yields, which was not done prior to culture. Perhaps as expected, PPT cultures identified pathogens not found on sonication fluid culture or sonication fluid sequencing; the sensitivity of sequencing of sonication fluid compared to the consensus species found combining sonication fluid and PPT cultures was only 68%. We also identified using sequencing four examples of probable anaerobic pathogens not identified by routine anaerobic culture of sonication fluid or PPT: Fusobacterium nucleatum, Veillonella parvula, Finegoldia magna, and Parvimonas micra. It is possible that these organisms may have been cultured had fastidious anaerobe agar been used as we used Columbia blood agar (CBA) plates for anaerobic culture, as previously described (7). We were also able to identify a plausible pathogen in two patients who had received prior antibiotics where the routine microbiology was uninformative.

Controlling for contamination during sampling and culture is a major challenge in investigating PJI and underlies why using multiple independent PPT samples remains the gold standard for diagnosis. Contamination is an even greater concern in molecular diagnostic assays, including metagenomic sequencing, given the additional potential for DNA contamination. There are published reports demonstrating the potential for contamination leading to misinterpretation of sequencing data from clinical specimens (34, 35). In our laboratory, samples were handled in laminar flow hoods and extracted in a dedicated pre-PCR extraction laboratory. DNA was handled in a PCR hood, and sequencing libraries were manipulated in a dedicated post-PCR sequencing laboratory. Despite these measures, we still observed contamination in some of our samples. During the derivation phase of our study, it is likely that one or more of the reagents used became contaminated with DNA from other sequencing projects in our laboratory. Although we were able to account for this in our analysis and then validate our findings in a separate set of samples having addressed this specific form of contamination, contamination remained a concern during the validation phase, as evidenced by an adjusted specificity of only 89% and by contamination of one of the negative controls leading to a batch of samples being discarded. This demonstrates that rigorous laboratory practice would be key to deploying our method. There may also be a role for sealed systems that perform DNA extraction and sequencing in a separated environment. Our experience also reinforces the requirement that negative controls are included in each sequencing batch, as is routine in molecular microbiology diagnostic assays, to ensure that contamination is detected if it does occur. A limitation of our study is that the saline used for sonication was not PCR grade, and this could be considered in future work.

Excluding the specific issue of contamination by other sequencing projects, P. acnes was the most common apparent contaminant. It affected one of the negative controls during the validation phase, and, overall, false-positive results for P. acnes were found in 7% of samples. Species-specific filtering may be required to address this; our one true-positive sample with P. acnes present on culture had >10^5^ P. acnes reads. However, larger data sets are required than ours to address this definitively. In the meantime, even with molecular diagnostics, the value of multiple samples per patient remains.

Sonication fluid can be a large-volume sample, typically 50 to 400 ml. As a result, the microbial cells released from the orthopedic device during sonication are likely to be heavily diluted. This, coupled with the simultaneous release of any human cells from the prosthesis and transfer of blood along with the device, results in a sonication fluid sample that is both low in bacterial cells and high in contaminating host cells. An effective microbial DNA extraction protocol is necessary to isolate as much bacterial DNA as possible while limiting the amount of host DNA in the final extract. Our results demonstrate that despite efforts to filter out human cells or remove human DNA postextraction, host DNA accounted for >90% of reads in the majority of samples sequenced. Use of a specialist microbiome enrichment kit did not improve bacterial DNA yield. However, if the efficiency of human DNA removal can be improved in the future, this might significantly add to the precision of metagenomic sequencing as more sequencing efforts would be appropriately directed toward potential pathogens.

In addition to the issues around contamination with bacterial and human DNA, a further limitation of our study as designed is that it undertakes a laboratory-level comparison of sonication fluid culture and metagenomics sequencing. As this study was conducted as laboratory method development, we made use of information available to the microbiology laboratory only at the time of sampling and did not review patient notes, and so we were unable to compare sonication fluid sequencing to the presence of a final overall diagnosis of infection. Future studies should consider how sequencing might contribute to the overall diagnosis of PJI as part of an assessment that jointly considers clinical, histological, and microbiological data.

This study demonstrates as a proof of principle that metagenomic sequencing can be used in the culture-free diagnosis of PJI directly from sonication fluid. Improvements to the method of human DNA removal from direct samples before sequencing are ongoing, and if these are successful, this is likely to greatly improve the efficiency, and therefore accuracy, of metagenomic sequencing. Generating greater numbers of bacterial reads directly from clinical specimens may make prediction of antimicrobial susceptibilities directly from samples possible, as has been achieved from whole-genome sequencing of cultured organisms (25–28). If this can be achieved reliably and if contamination from human and other bacterial DNA can be minimized, it is possible that sequencing can offer a complete microbiology diagnosis without the need for culture. The increasing availability of portable, rapid, random-access strand sequencing technology offers the potential that in the future sequencing may become a same-day diagnostic tool. Applications of rapid sequencing in PJI might include perioperative microbiological diagnosis to guide the use of local intraoperative antimicrobials, for example, in cement or beads. Earlier diagnosis may also ensure that postoperative antimicrobials are more focused, improving antimicrobial stewardship, while treating resistant organisms effectively. Earlier diagnosis may also reduce hospital stays and therefore reduce costs. Sequencing is also likely to be helpful in situations where multiple samples containing the same commensal species are identified. Sequencing will be able to determine whether these are clonal, suggesting true infection rather than contamination, instead of having to rely on current proxies such as antimicrobial susceptibility profiles, which only imperfectly distinguish nonclonal isolates. Ultimately, same-day sequencing may significantly improve the precision, efficiency, and cost of PJI care. This study provides a foundation for further development toward this goal.

## MATERIALS AND METHODS

### Sample collection and processing.

Intraoperative samples from the Nuffield Orthopaedic Centre (NOC) in Oxford University Hospitals (OUH), United Kingdom, between June 2013 and January 2017 were investigated. The NOC is a tertiary-level specialist musculoskeletal hospital, including a dedicated Bone Infection Unit, undertaking approximately 200 revision arthroplasties annually. A subset of samples submitted was chosen at random following culture to provide a ratio of approximately 2:1 bacterial culture-positive samples to culture-negative samples. For this study, no ethical review was required, because the study was a laboratory method development study focusing on bacterial DNA extracted from discarded samples identified only by laboratory numbers, with no personal or identifiable data. Sequencing reads identified as human on the basis of Kraken were counted and immediately permanently discarded.

Prosthetic joint implants and metalwork, received into the OUH microbiology laboratory following revision arthroplasty and operative management of other orthopedic device-related infection, were placed directly into single-use sterile polypropylene containers (Lock & Lock brand) and covered with between 10 ml and 400 ml of sterile 0.9% saline solution (Oxoid, Ltd., Basingstoke, United Kingdom) depending on the size of the prosthesis/device, with sufficient fluid to cover at least 90% of the prosthesis/device, up to a maximum of 400 ml. Sonication was performed as described previously (7) with minor modifications. Briefly, the implant was vortexed for 30 s, subjected to sonication for 1 min, followed by additional vortexing for 30 s. Sonication was performed in a Bransonic 5510 ultrasonic water bath (Branson, Danbury, CT, USA) at a frequency of 40 kHz. The resulting sonication fluid was plated in 0.1-ml aliquots onto Columbia blood agar (CBA) and chocolate agar plates (Oxoid, Ltd., Basingstoke, United Kingdom) for aerobic incubation and on CBA plates for anaerobic incubation. Aerobic incubation was performed at 35 to 37°C with 5% CO_2_ for up to 5 days. Anaerobic incubation was performed at 35 to 37°C for 10 days. All cultured microorganisms were identified by matrix-assisted laser desorption ionization–time of flight (MALDI-TOF) mass spectrometry on a Microflex LT using Biotyper, version 3.1 (Bruker Daltonics, Billerica, MA, USA). Samples were considered culture positive when growth of ≥50 CFU/ml was observed and additionally when growth of a highly pathogenic organism (including Staphylococcus aureus and Enterobacteriaceae) at <50 CFU/ml was observed.

Periprosthetic tissue samples were also collected during surgery, at the start of each procedure and using different surgical instruments for each sample, and processed by the microbiology laboratory. Briefly, Bactec bottles were inoculated with 0.5 ml of an inoculum generated by vortexing each tissue sample in 3 ml of 0.9% saline with sterile Ballotini balls for 15 s. Bottles were incubated under aerobic (Plus Aerobic/F culture vials) and anaerobic (Lytic/10 Anaerobic/F culture vials) conditions in a BD Bactec FX system (BD Biosciences, Sparks, MD, USA) for up to 10 days. Any bottles that flagged positive were subcultured onto agar plates and processed as described above to determine species.

### Bacterial DNA extraction from sonication fluid.

Prior to DNA extraction, sonication fluids were concentrated by centrifugation. Forty milliliters of fluid was transferred to a sterile, disposable 50-ml polypropylene tube and centrifuged at 15,000 × *g* in a Sorvall RC5C Plus centrifuge (SLA-1500 rotor with custom-made inserts) for 1 h at 16°C. Samples with a <40-ml starting volume of sonication fluid were made up to 40 ml with the same saline used for sonication. All but approximately 1 ml of the supernatant was discarded, and the pellet was resuspended in this volume of fluid before being passed through a 5-μm-pore-size syringe filter to deplete the number of human cells present and, therefore, the amount of human DNA in the final extract. Bacterial cells passing through the filter were pelleted, washed with 1 ml of 0.9% saline, and resuspended in 500 μl of molecular-biology-grade water before being mechanically lysed in Pathogen Lysis tubes (S) (Qiagen, Hilden, Germany) with a FastPrep 24 tissue homogenizer (MP Biomedicals, Santa Ana, CA, USA) (three times for 40 s at 6.5 m/s). DNA was extracted by ethanol precipitation, using GlycoBlue (Life Technologies, Paisley, UK) as a coprecipitant, and resuspended in 50 μl of 1× Tris-EDTA (TE) buffer. DNA was purified using AMPure XP solid-phase reversible immobilization (SPRI) beads (Beckman Coulter, High Wycombe, United Kingdom) and eluted in 26 μl of TE buffer. DNA concentration was measured using a Qubit 2.0 fluorometer (Life Technologies, Paisley, United Kingdom). A subset of samples was treated with an NEBNext microbiome DNA enrichment kit (New England BioLabs, Ipswich, MA, USA) for human DNA removal before an additional purification step using AMPure XP SPRI beads and final elution in 15 μl of TE buffer. Samples were extracted in batches, with a negative control of sterile 0.9% saline prepared alongside each batch using this same protocol.

### Library preparation and Illumina MiSeq sequencing.

DNA extracts quantified as ≥0.2 ng/μl were sequenced on a MiSeq desktop sequencer (Illumina, San Diego, CA, USA). Libraries were prepared as previously described, using a variation of the Illumina Nextera XT protocol (36). Briefly, 1 ng of DNA was prepared for sequencing following the Illumina Nextera XT protocol, with the modification of 15 cycles during the index PCR. Libraries were quantified using a Qubit 2.0 fluorometer, and their average sizes were determined with an Agilent 2200 TapeStation (Agilent Technologies, Santa Clara, CA, USA) before being manually normalized. Libraries were prepared and sequenced together in the same batch. Paired-end sequencing was performed using a 600-cycle MiSeq reagent kit (version 3), and samples were sequenced in batches of between 1 and 13 on a single flow cell.

### Bioinformatics analysis.

Raw sequencing reads were adapter trimmed using BBDuk (https://sourceforge.net/projects/bbmap/) and the adapter sequence file provided within the BBMap package; the following parameters were used: minlength, 36; k,19; ktrim, r; hdist, 1; mink, 12. Taxonomic classification of trimmed reads was performed using Kraken (37) and a bespoke database constructed from all bacterial genomes deposited in the NCBI RefSeq database as of January 2015 (updated January 2017 for the validation set; see below), with default parameters and no k-mer removals. Where no RefSeq genome was available for an organism cultured from a PJI at OUH since June 2013, available whole-genome assemblies were also added to the database where available in NCBI. Additionally, the Genome Reference Consortium Human genome build 38 (GRCh38) was included in the database to allow detection of host DNA. An optimum filtration threshold, using a Kraken filter that balanced false-positive removal and sensitivity, was determined using simulated data sets of reference genomes. Reference genomes representative of common pathogenic species were used to generate simulated Illumina MiSeq data sets and analyzed with Kraken using different filtration thresholds. A threshold value of 0.15 provided optimum read classification sensitivity while minimizing spurious results. Kraken output was visualized using Krona (38).

### Statistical analysis.

The performance of metagenomic sequencing was assessed by comparing the species identified from sequencing data with the species isolated from sonication fluid samples considered culture positive (i.e., ≥50 CFU/ml or growth of a highly pathogenic organism at <50 CFU/ml). In order to correct for samples which may contain small numbers of contaminating and nonspecific bacterial reads, a threshold was determined to identify the presence of true infection, using the first 50 samples sequenced as a derivation set. Two thresholds (1 and 2), and three parameters (a to c), were used to determine true infection: (i) samples with more reads from a given species than an upper-read cutoff (a) were included; (ii) samples with more species-specific reads than a lower-read cutoff (b) and with the percentage of species-specific reads as a proportion of all bacterial reads present above a percentage cutoff (c) were also included. Parameter values were selected by numerical optimization, using R, version 3.3.2, comparing sequencing results to sonication fluid culture results and maximizing the value of the Youden index (39) (sensitivity + specificity − 1). Sensitivity was calculated taking each species identified from each culture-positive sonication sample as a separate data point; thus, culture-negative samples did not contribute to the denominator, culture-positive samples with a single species contributed once, and culture-positive samples with two species contributed twice. Specificity was calculated using the total number of sonication samples as the denominator; as such samples contaminated by more than one species were counted as one false positive.

To ensure that read cutoff parameters were chosen without a penalty for potentially difficult to culture anaerobic species, the specificity value optimized was adjusted. Potential false-positive sequencing results with plausible fastidious anaerobic causes of infection (including Fusobacterium nucleatum, Propionibacterium acnes, and Veillonella parvula) in culture-negative samples were excluded when the specificity value used for parameter optimization was calculated.

Where bacterial reads were detected over the thresholds described above in a negative control, that sample was deemed to be contaminated. In the derivation set, in order to maximize the number of sequences available for analysis, only samples with evidence of the same contaminating organisms were excluded from each contaminated batch, rather than discarding the whole batch. During the derivation phase of the study, several batches of samples were found to be contaminated with DNA from other studies performed concurrently in the same research laboratory. Six of eight saline negative-control extracts displayed contamination with a single or multiple species at read numbers exceeding the determined diagnostic thresholds. All samples within these batches that displayed similar contamination levels were excluded from subsequent analysis if Kraken classification resulted in >100 reads corresponding to the majority of the contaminating species. A total of 22 samples (in addition to the 50 successfully sequenced) were excluded on this basis (Fig. 1). In batches 4 and 5 the negative controls were contaminated with Staphylococcus aureus, Escherichia coli, and P. acnes, and 15 samples were excluded with >100 reads from ≥2/3 species; in batch 6 the negative control was contaminated with Serratia marcescens, Klebsiella pneumoniae, E. coli, and P. acnes, and 2 samples with >100 reads from ≥3/4 species were excluded; in batches 2, 9, and 10 the negative control was contaminated with P. acnes, and 5 samples were excluded with >100 P. acnes reads. To address this issue, prior to the validation phase of the study, all pipettes, laminar flow and PCR hoods, and laboratory benches used for DNA extraction and library preparation were deep-cleaned with Virkon disinfectant and RNase Away surface decontaminant (Thermo Fisher Scientific, Waltham, MA, USA) in order to remove any possible sources of microbial or DNA contamination. All DNA extraction and library preparation reagents were replaced and used in preprepared per-batch aliquots used exclusively for this study. Sonication fluid samples were handled one at a time in the laminar flow hood, which was cleaned as above between each sample. Fresh gloves were worn each time a new sample was handled during the DNA extraction phase of the protocol. Having implemented these changes, for the validation phase, a more stringent quality control standard was applied, requiring the negative control to be contamination free for any of the samples in a batch to be analyzed.

### Technical replicates.

To ensure sequencing reproducibility, one DNA sample was sequenced twice, and biological replicates (DNA extraction process repeated) were sequenced for six samples (four in duplicate and two in triplicate).

## Supplementary Material

Supplemental material
