# Supplementary material for: Molecular Diagnosis of Orthopedic-Device-Related Infection Directly from Sonication Fluid by Metagenomic Sequencing
Source: J Clin Microbiol. 2017 Jul 25;55(8):2334–47. doi: 10.1128/JCM.00462-17 (PMC5527411; doi:10.1128/JCM.00462-17)
Supplement: Supplemental material [file supp_55_8_2334__index.html]

Supplemental material 

# Molecular Diagnosis of Orthopedic-Device-Related Infection Directly from Sonication Fluid by Metagenomic Sequencing

## Supplemental material

- Supplemental file 1 -

  Tables S1 (Additional information for all samples passing thresholds for analysis in the derivation and validation data sets) and S2 (Comparison of human read numbers observed in a subset of samples treated with or without the NEBNext microbiome DNA enrichment kit)

  PDF, 522K
