# Supplementary material for: Molecular Diagnosis of Orthopedic-Device-Related Infection Directly from Sonication Fluid by Metagenomic Sequencing
Source: J Clin Microbiol. 2017 Jul 25;55(8):2334–47. doi: 10.1128/JCM.00462-17 (PMC5527411; doi:10.1128/JCM.00462-17)
Supplement: Supplemental material [file JCM.00462-17_zjm999095578s1.pdf]

Molecular diagnosis of orthopaedic device infection direct from sonication fluid by metagenomic sequencing: Supplemental material

Sample	Sonication species	Sonication CFU	Sequencing species	Reads	% Bacterial reads	Tissue culture species	Tissue samples (positive/total)	Consensus pathogen(s)	Concordance with sequencing	Total reads	Human reads	Bacterial reads	Joint	Sonication fluid volume
**Derivation Set**														
**164e**	*Staphylococcus epidermidis*	>490	*Staphylococcus epidermidis*	2716	81%	*Staphylococcus epidermidis*	3/5	*S. epidermidis*	Complete	4620748	4616783	3352	Knee	400
**171c**	*Staphylococcus epidermidis*	>490	*Staphylococcus epidermidis*	3154	79%	*Staphylococcus epidermidis*	2/5	*S. epidermidis*	Complete	3484832	3479994	3976	Hip	100
**176e**	No growth					*Staphylococcus aureus*	1/4	*S. aureus, P. aeruginosa*	No	3894599	3893752	120	Hip	200
						Pseudomonas aeruginosa	3/4							
**182c**	*Enterococcus faecium*	100-240	*Enterococcus faecium*	144	43%	*Enterococcus faecium*	6/6	*E. faecium*	Complete	4289791	4288758	338	Knee	300
**183d**	*Staphylococcus epidermidis*	>490	*Staphylococcus epidermidis*	3362	87%	*Staphylococcus epidermidis*	4/5	*S. epidermidis*	Complete	3289466	3285103	3869	Hip	200
**193c_d2**	*Staphylococcus aureus*	>490	*Staphylococcus aureus*	360718	97%	*Staphylococcus aureus*	5/5	*S. aureus*	Complete	27605320	27222536	370648	Knee	200
	Staphylococcus condimenti	>490												
**198c**	*Staphylococcus epidermidis*	>490	*Staphylococcus epidermidis*	228	52%	*Staphylococcus epidermidis*	3/5	*S. epidermidis*	Complete	2828797	2827907	439	Knee	400
**208a**	*Enterococcus faecalis*	>490	*Enterococcus faecalis*	14486	31%	*Enterococcus faecalis*	5/5	*E. faecalis, E. coli*	Complete	2910881	2864109	46214	Hip	100
	Escherichia coli	250-490	*Escherichia coli*	6503	14%	*Escherichia coli*	4/5							
**213a**	*Staphylococcus aureus*	>490	*Staphylococcus aureus*	167	80%	*Staphylococcus aureus*	5/5	*S. aureus*	Complete	2599456	2598429	208	Metalwork (femur)	25
**219a**	*Staphylococcus lugdunensis*	>490	*Staphylococcus lugdunensis*	411	27%	*Staphylococcus lugdunensis*	3/4	*S. lugdunensis, C. propinquum*	Partial	2331579	2329646	1524	Metalwork (tibia)	100
			Achromobacter xylosoxidans	722	47%	*Corynebacterium propinquum*	4/4							
						Staphylococcus epidermidis	1/4							
**223a**	*Staphylococcus aureus*	>490	*Staphylococcus aureus*	7504	95%	*Staphylococcus aureus*	4/5	*S. aureus*	Complete	2227131	2218173	7879	Knee	100
**229a**	*Staphylococcus aureus*	>490	*Staphylococcus aureus*	6038	98%	*Staphylococcus aureus*	1/2	*S. aureus*	Complete	1067494	1060914	6182	Hip	100
**249a**	*Propionibacterium acnes*	>490	*Propionibacterium acnes*	108940	100%	*Propionibacterium acnes*	4/5	*P. acnes*	Complete	2652741	2542883	109382	Hip	200
**259a**	*Staphylococcus epidermidis*	>490	*Staphylococcus epidermidis*	749	86%	*Staphylococcus epidermidis*	3/4	*S. epidermidis*	Complete	2502322	2501038	869	Knee	400
**289a**	*Staphylococcus aureus*	250-490	*Staphylococcus aureus*	2105	94%	*Staphylococcus aureus*	5/5	*S. aureus*	Complete	1755708	1752782	2251	Hip	200

**296a**	*Serratia marcescens*	250-490	*Serratia marcescens*	590	60%	*Serratia marcescens*	4/4	*S. marcescens*	Complete	2140250	2138792	986	Knee	300
**312a**	*Citrobacter koseri*	>490	*Citrobacter koseri*	221516	95%	*Citrobacter koseri*	4/5	*C. koseri*	Complete	2578017	2343324	234334	Hip	200
**329a**	*Morganella morganii*	>490	*Morganella morganii*	18553	95%	*Morganella morganii*	6/6	*M. morganii*	Complete	2483844	2463778	19631	Knee	100
**335a**	*Morganella morganii*	100-240	*Morganella morganii*	3555	94%	*Morganella morganii*	3/5	*M. morganii*	Complete	3185564	3181313	3800	Knee	400
**341a**	No growth		*Staphylococcus aureus*	153	42%	No growth	0/3	None identified	Sequencing potentially additive	2684434	2683596	368	Metalwork (foot)	20
**346a**	No growth					*Staphylococcus aureus*	3/5	*S. aureus, M. fortuitum*	No	3521279	3520619	89	Knee	400
						Mycobacterium fortuitum	1/5							
**352a**	*Bacillus* species	100-240	*Bacillus* species	1110		*Bacillus* species	2/5	*Bacillus spp.*	Partial	2934240	2932243	1281	Hip	100
**354a**	*Arcanobacterium haemolyticum*	>490	*Arcanobacterium haemolyticum*	11182	72%	*Staphylococcus aureus*	2/6	Extensive polymicrobial infection	Partial	3087375	3071048	15506	Hip	200
	Enterococcus faecalis	>490	*Enterococcus faecalis*	1173	8%	*Enterococcus faecalis*	4/6							
			Fusobacterium nucleatum	1156	7%	*Streptococcus oralis*	1/6							
						CoNS	5/6							
						Pseudomonas aeruginosa	1/6							
						Corynebacterium striatum	1/6							
						Staphylococcus epidermidis	2/6							
**358a**	No growth					No growth	0/3	None identified	Complete	1950760	1950316	50	Ankle	100
**359a**	No growth		*Propionibacterium acnes*	464	24%	*Staphylococcus epidermidis*	1/4	None identified	No	471382	469370	1919	Shoulder	400
**361a**	*Finegoldia magna*	>490	*Finegoldia magna*	3674	95%	No growth	0/6	*F. magna*	Complete	2163209	2158886	3849	Spine	100
**362a**	*Propionibacterium acnes*	<50				No growth	0/1	None identified	Complete	1949195	1948689	90	Knee	300
**364a**	No growth					No growth	0/4	None identified	Complete	1640324	1639833	242	Knee	400
**365a**	No growth		*Propionibacterium acnes*	318	23%	No growth	0/1	None identified	No	2145032	2143226	1389	Hip	400
**366a**	*Klebsiella pneumoniae*	>490	*Klebsiella pneumoniae*	8981	25%	*Klebsiella pneumoniae*	4/5	*K. pneumoniae*	Complete	1654489	1617772	35844	Knee	100
**368a**	No growth		*Ralstonia pickettii*	3146	40%	No growth	0/4	None identified	No	3446730	3437856	7956	Hip	400
			Enterobacter cloacae	2629	33%									
**369a**	*Enterobacter cloacae*	>490	*Enterobacter cloacae*	2502	11%	*Enterobacter cloacae*	4/5	*E. cloacae, P. aeruginosa, S.*	Partial	1661953	1638790	22786	Ankle	100

	Pseudomonas aeruginosa	100-240	*Pseudomonas aeruginosa*	1192	5%	*Pseudomonas aeruginosa*	5/5	*epidermidis*						
			Veillonella parvula	14801	65%	*Staphylococcus epidermidis*	4/5							
						Staphylococcus lugdunensis	1/5							
**370a**	*Propionibacterium acnes*	<50				No growth	0/4	None identified	Complete	3645259	3644089	54	Knee	Not recorded
**371a**	*Staphylococcus epidermidis*	>490	*Staphylococcus epidermidis*	4998	87%	*Staphylococcus epidermidis*	3/3	*S. epidermidis*	Complete	3665161	3658321	5761	Hip	Not recorded
						CoNS	1/3							
**372a**	No growth		*Propionibacterium acnes*	3874	51%	*S. aureus*	4/4	*S. aureus*	No	1100377	1092496	7584	Metalwork (femur)	100
						G. adiacens	1/4							
**373a**	*Enterococcus faecalis*	>490	*Enterococcus faecalis*	1234	38%	*Enterococcus faecalis*	1/5	*E. faecalis, S. epidermidis*	Complete	4421920	4417533	3271	Knee	400
	Staphylococcus epidermidis	100-240	*Staphylococcus epidermidis*	616	19%	*Staphylococcus epidermidis*	3/5							
**374a**	No growth					No growth	0/4	None identified	Complete	2979956	2979093	265	Hip	Not recorded
**375a**	No growth		*Propionibacterium acnes*	5686	75%	*S. epidermidis*	1/5	None identified	No	2558853	2543040	7540	Hip	25
**376a**	*Enterobacter cloacae*	>490	*Enterobacter cloacae*	122622	95%	*Enterobacter cloacae*	4/4	*E. cloacae*	Complete	2301591	2170696	128909	Knee	Not recorded
	Coagulase-negative *Staphylococcus*	>490												
**379a**	No growth					*Staphylococcus aureus*	3/5	*S. aureus*	No	4765021	4763828	47	Knee	400
						CoNS	1/5							
**382a**	*Staphylococcus aureus*	<50	*Staphylococcus aureus*	440	50%	*Staphylococcus aureus*	4/4	*S. aureus, S. dysgalactiae*	Partial	1785164	1783211	882	Hip	200
						Streptococcus dysgalactiae	2/4							
**383a**	No growth					No growth	0/4	None identified	Complete	2353538	2352976	83	Metalwork (femur)	200
**384a**	*Staphylococcus epidermidis*	>490	*Staphylococcus epidermidis*	1751	85%	*Staphylococcus epidermidis*	2/4	*S. epidermidis*	Complete	2811486	2805957	2067	Knee	Not recorded
**388a**	No growth					No growth	0/3	None identified	Complete	2197885	2197214	207	Knee	Not recorded
**389a**	No growth					*Staphylococcus epidermidis*	2/5	*S. epidermidis*	No	3146355	3141733	173	Hip	400
						Bacillus species	1/5							
**391a**	No growth					No growth	0/4	None identified	Complete	2169982	2167908	59	Hip	200
**399a**	*Staphylococcus aureus*	Not recorded	*Staphylococcus aureus*	1955	97%	*Staphylococcus aureus*	2/5	*S. aureus*	Complete	2298339	2295545	2022	Knee	400

**404a**	*Staphylococcus aureus*	>490	*Staphylococcus aureus*	2257	39%	*Staphylococcus aureus*	4/6	*S. aureus, C. striatum, E. coli*	Partial	1733153	1726743	5854	Hip	400
						Corynebacterium striatum	5/6							
						Escherichia coli	2/6							
**408a**	*Staphylococcus aureus*	>490	*Staphylococcus aureus*	368	87%	*Staphylococcus aureus*	4/4	*S. aureus*	Complete	1722950	1721536	425	Knee	Not recorded
**410a**	*Staphylococcus aureus*	100-240	*Staphylococcus aureus*	235	27%	*Staphylococcus aureus*	4/4	*S. aureus*	Partial	1998806	1997229	870	Metalwork (ankle)	15
			Corynebacterium jeikeium	401	46%	CoNS	1/4							
**Validation Set**														
**256a**	*Gemella morbillorum*	>490	*Gemella morbillorum*	784	72%	*Gemella morbillorum*	6/6	*G. morbillorum*	Complete	2153943	2153429	323	Knee	400
**397a**	*Staphylococcus epidermidis*	>490	*Staphylococcus epidermidis*	6717	94%	*Staphylococcus epidermidis*	5/5	*S. epidermidis*	Complete	2184267	2176942	7131	Knee	400
**400a**	*Aeromonas hydrophila*	>490				*Aeromonas species*	3/4	*P. aeruginosa, A. hydrophila E. faecalis, S. aureus, K. oxytoca*	Partial	2646321	2002366	635860	Metalwork (tibia)	Not recorded
	Staphylococcus aureus	100-240	*Staphylococcus aureus*	6547	5%	*Staphylococcus aureus*	4/4							
			Pseudomonas aeruginosa	86920	68%	*Pseudomonas aeruginosa*	2/4							
			Klebsiella oxytoca	1238	1%	*Klebsiella oxytoca*	1/4							
			Finegoldia magna	15606	12%									
			Enterococcus faecalis	1303	1%	*Enterococcus faecalis*	1/4							
**405a**	*Staphylococcus lugdunensis*	>490	*Staphylococcus lugdunensis*	311	96%	*Staphylococcus lugdunensis*	6/6	*S. lugdunensis*	Complete	2142037	2141545	325	Hip	Not recorded
**406a**	*Enterococcus faecium*	250-490				*Enterococcus faecium*	2/3	*E. faecium*	No	2136191	2135982	16	Knee	200
**409a**	*Streptococcus agalactiae*	>490	*Streptococcus agalactiae*	2556	93%	*Streptococcus agalactiae*	5/5	*S. agalactiae*	Complete	2508772	2505789	2762	Knee	300
**423a**	*Staphylococcus aureus*	>490	*Staphylococcus aureus*	15479	98%	*Staphylococcus aureus*	4/4	*S. aureus*	Complete	1950278	1933719	15859	Metalwork (femur)	100
**426a**	*Staphylococcus aureus*	250-490	*Staphylococcus aureus*	11981	89%	*Staphylococcus aureus*	2/4	*S. aureus*	Complete	2438334	2424048	13518	Metalwork (Ulna)	100
**430a**	*Streptococcus pneumoniae*	>490	*Streptococcus pneumoniae*	5697	82%	*Streptococcus pneumoniae*	5/5	*S. pneumoniae*	Complete	2005371	1998239	6949	Hip	400
**442a**	*Enterococcus faecium*	>490	*Enterococcus faecium*	1689	68%	*Enterococcus faecium*	5/5	*E. faecium*	Complete	2260887	2258127	2502	Knee	400
**450a**	*Staphylococcus aureus*	>490	*Staphylococcus aureus*	2584	98%	*Staphylococcus aureus*	5/6	*S. aureus*	Complete	1978198	1972554	5300	Hip	100
						Propionibacterium spp.	1/6							
**459a**	*Streptococcus agalactiae*	>490	*Streptococcus agalactiae*	114212	93%	*Streptococcus agalactiae*	5/5	*S. agalactiae*	Complete	2702329	2579158	122911	Knee	400

**465a**	*Staphylococcus aureus*	>490	*Staphylococcus aureus*	1171	97%	*Staphylococcus aureus*	4/4	*S. aureus*	Complete	1739217	1737841	1204	Knee	100
**468a**	*Staphylococcus aureus*	>490	*Staphylococcus aureus*	676	93%	*Staphylococcus aureus*	3/3	*S. aureus*	Complete	2052578	2051663	729	Knee	200
**473a**	*Enterococcus faecalis*	250-490	*Enterococcus faecalis*	228	73%	*Enterococcus faecalis*	4/4	*E. faecalis*	Complete	1650196	1649744	306	Hip	400
**474a**	*Staphylococcus epidermidis*	250-490				*Corynebacterium striatum*	2/5	*C. striatum, S. aureus, S. epidermidis*	Partial	2080013	2078141	477	Hip	400
						Staphylococcus aureus	1/5							
						Staphylococcus epidermidis	3/5							
**480a**	*Staphylococcus epidermidis*	250-490	*Staphylococcus epidermidis*	557	80%	*Staphylococcus epidermidis*	5/5	*S. epidermidis*	Complete	123953	123242	698	Hip	400
**482a**	*Staphylococcus epidermidis*	>490	*Staphylococcus epidermidis*	1327	88%	*Staphylococcus epidermidis*	5/5	*S. epidermidis*	Complete	2097975	2096139	1502	Knee	200
**483a**	*Staphylococcus aureus*	100-240	*Staphylococcus aureus*	444	85%	No growth	0/5	*S. aureus*	Complete	3041395	3040460	520	Knee	200
**485a**	*Gemella morbillorum*	>490	*Gemella morbillorum*	123300	18%	*Gemella morbillorum*	3/4	Extensive polymicrobial infection	Partial	1119282	942412	176105	Hip	400
			Parvimonas micra	508822	76%	*Streptococcus oralis*	1/4							
			Streptococcus equi	16580	2%	*Staphylococcus aureus*	1/4							
			Streptococcus anginosus	8019	1%	*Corynebacterium amycolatum*	1/4							
						Proteus mirabilis	1/4							
**486a**	*Enterococcus faecalis*	>490	*Enterococcus faecalis*	3904	43%	*Enterococcus faecalis*	5/5	*E. faecalis, S. epidermidis*	Partial	3095177	3076395	17962	Knee	100
						Staphylococcus epidermidis	4/5							
**487a**	*Staphylococcus aureus*	<50	*Staphylococcus aureus*	121284	98%	*Staphylococcus aureus*	2/4	*S. aureus*	Complete	2068841	1940883	123535	Shoulder	300
**489a**	*Staphylococcus aureus*	100-240	*Staphylococcus aureus*	858	95%	*Staphylococcus aureus*	2/4	*S. aureus*	Complete	2096505	2094141	1800	Knee	400
						Staphylococcus epidermidis	1/4							
**498a**	*Staphylococcus aureus*	<50	*Staphylococcus aureus*	135	88%	*Staphylococcus aureus*	4/5	*S. aureus*	Complete	2499995	2499535	154	Knee	400
**504a**	*Staphylococcus aureus*	>490	*Staphylococcus aureus*	3229	97%	*Staphylococcus aureus*	7/7	*S. aureus*	Complete	2663338	2659560	3340	Knee	Not recorded
**507a**	*Proteus mirabilis*	<50	*Proteus mirabilis*	184	15%	*Proteus mirabilis*	2/5	*P. mirabilis*	Partial	2408063	2406496	1188	Knee	100
			Morganella morganii	981	83%									
**511a**	*Pseudomonas aeruginosa*	Not recorded				*Pseudomonas aeruginosa*	3/6	*P. aeruginosa*	No	2243128	2240415	2006	Metalwork (Tibia)	100
			Propionibacterium acnes	1377	69%									
**513a**	*Citrobacter spp.*	<50	*Citrobacter koseri*	1133	87%	*Citrobacter* species	2/5	*C. koseri*	Complete	2321321	2319644	1299	Knee	100

**514a**	*Staphylococcus epidermidis*	>490	*Staphylococcus epidermidis*	11803	91%	*Staphylococcus epidermidis*	5/5	*S. epidermidis*	Complete	2534764	2521530	12903	Hip	80
**516a**	Coagulase-negative *Staphylococcus*	100-240				No growth	0/4	None identified	Complete	1761591	1761409	27	Shoulder	100
**414a**	*Staphylococcus epidermidis*	<50	*Staphylococcus epidermidis*	1194	91%	*Staphylococcus epidermidis*	5/5	*S. epidermidis*	Complete	1951636	1950159	1316	Knee	200
**472a**	No growth					No growth	0/4	None identified	Complete	2187974	2187626	85	Hip	400
**475a**	No growth		*Streptococcus dysgalactiae*	156	37%	*Micrococcus luteus*	1/4	None identified	Sequencing potentially additive	2329604	2328896	427	Metalwork (Foot)	25
**476a**	No growth					*Pseudomonas aeruginosa*	4/4	*P. aeruginosa, S. aureus*	No	41535	40953	558	Ankle	100
						Staphylococcus aureus	3/4							
**478a**	No growth					Coagulase-negative *Staphylococcus*	1/4	None identified	Complete	2516669	2516389	7	Knee	400
**490a**	*Staphylococcus epidermidis*	<50				No growth	0/5	None identified	Complete	2005147	2004977	9	Ankle	100
**496a**	No growth					*Bacillus* species	1/4	None identified	Complete	38857	38480	362	Ankle	100
**497a**	*Streptococcus vestibularis*	<50				*Corynebacterium striatum*	4/4	*C. striatum*	No	2630484	2630047	26	Metalwork (humerus)	100
**502a**	No growth		*Corynebacterium aurimucosum*	2379	42%	*Corynebacterium aurimucosum*	2/4	*E. faecium,* Coagulase-negative *Staphylococcus, C. aurimucosum, S. epidermidis*	Partial	3018683	2994803	22688	Hip	200
			Staphylococcus epidermidis	1336	24%	*Staphylococcus epidermidis*	4/4							
						Enterococcus faecium	3/4							
						Coagulase-negative *Staphylococcus*	2/4							
**503a**	*Coagulase-negative Staphylococcus*	<50				No growth	0/5	None identified	Complete	1577886	1577569	196	Ankle	100
**505a**	No growth					No growth	0/5	None identified	Complete	1742544	1742397	27	Hip	Not recorded
**506a**	No growth					No growth	0/6	None identified	Complete	2450468	2450085	38	Spine (screws)	100
**508a**	No growth					No growth	0/8	None identified	Complete	2367559	2365792	1374	Knee	200
**509a**	No growth					No growth	0/5	None identified	Complete	2694622	2694249	59	Hip	100
**510a**	No growth		*Staphylococcus epidermidis*	290	26%	*Staphylococcus epidermidis*	2/4	*S. epidermidis*	Partial	933953	932686	1121	Knee	400
			Propionibacterium acnes	232	21%									

**512a**	*Staphylococcus epidermidis*	<50				*Staphylococcus epidermidis*	5/5	*S. epidermidis*	No	2288713	2288269	126	Knee	Not recorded
**515a**	No growth		*Propionibacterium acnes*	873	34%	*Enterococcus faecalis*	1/7	*E. faecalis*	No	24473	21899	2558	Hip	100

TABLE S1: Additional sample information giving full genus names; consensus microbiology diagnosis; concordance between consensus diagnosis and sequencing results; total, human and bacterial read numbers; joint type; and sonication fluid volume for all samples passing thresholds for analysis in the derivation (*n*=50) and validation (*n*=47) data sets.

	**NEBNext kit treatment**		**No treatment**	
**Sample**	**Human reads**	**% total reads**	**Human reads**	**% total reads**
1	3545442	98.4	4236821	98.1
2	3479994	98.4	2319421	98.3
3	3893752	98.7	3985696	98.7
4	4288758	98.9	3677517	98.8
5	3285103	98.7	3381451	98.3
6	3278051	97.3	3079783	96.7

TABLE S2: Comparison of human read numbers observed in a subset of samples treated with or without the NEBNext Microbiome DNA Enrichment kit.
